# Anti-contactin-1 autoimmune nodopathy with thymoma: case report and literature review

**DOI:** 10.3389/fimmu.2025.1662299

**Published:** 2025-09-09

**Authors:** Takumi Tashiro, Hidenori Ogata, Motoi Kuwahara, Haruo Nishijima, Kenichiro Nogami, Takahiro Yamaguchi, Hitoshi Hayashida, Guzailiayi Maimaitijiang, Ryo Yamasaki, Jun-ichi Kira, Yoshitaka Nagai, Noriko Isobe

**Affiliations:** ^1^ Department of Neurology, Neurological Institute, Graduate School of Medical Sciences, Kyushu University, Fukuoka, Japan; ^2^ Department of Neurology, Kindai University Faculty of Medicine, Osaka, Japan; ^3^ Department of Neurology, Hirosaki University Hospital, Aomori, Japan; ^4^ Department of Neurology, National Hospital Organization Beppu Medical Center, Oita, Japan; ^5^ Division of Clinical Chemistry and Laboratory Medicine, Kyushu University Hospital, Fukuoka, Japan; ^6^ Translational Neuroscience Research Center, Graduate School of Medicine, International University of Health and Welfare, Fukuoka, Japan

**Keywords:** contactin-1, autoimmune nodopathy, thymoma, demyelinating neuropathy, biomarker

## Abstract

**Backgrounds:**

Autoimmune nodopathies (ANs) with autoantibodies against cell adhesion molecules including contactin-1 (CNTN1) located in the nodes of Ranvier and paranodes have specific clinical features. Patients with anti-CNTN1 AN accompanied by paraneoplastic status have been reported. Here, we describe three patients with anti-CNTN1 AN and concurrent thymoma. In addition, we performed a literature review to investigate the relationship between demyelinating peripheral neuropathy and thymoma.

**Methods:**

Serum CNTN1-IgG and IgG subclasses were measured by cell-based assay or enzyme-linked immunosorbent assay. Clinical, electrophysiological, and pathological data were obtained from the medical records of patients. CNTN1-IgG titers and serum neurofilament light chain (sNfL) were followed over the disease course. Through a literature survey of demyelinating peripheral neuropathy and thymoma, we summarized the clinical features and involvement of CNTN1-IgG.

**Results:**

Three patients with CNTN1-IgG4 were elderly men, who presented with subacute disease progression, sensory ataxia, very high cerebrospinal fluid (CSF) protein levels, and apparent conduction delay in nerve conduction studies. In two patients with type B3 thymoma, thymectomy did not improve their neurological symptoms. In one case with type AB thymoma, nephrotic syndrome and pemphigus foliaceus appeared with the deterioration of neuropathy after thymectomy. All patients responded well to immunotherapies in parallel with decreased antibody titers and sNfL levels. Our literature survey identified a total of twelve cases, including our patients, of demyelinating peripheral neuropathy with thymoma. Ten were male and the mean age at onset was 57 years. Limb weakness and sensory ataxia were observed in 73% and 71% of patients, respectively. CSF protein levels were elevated in seven of nine patients. Four patients with Good’s syndrome or malignant thymoma progressed to death, whereas the other patients had a favorable response to mono- or combined immunotherapies. CNTN1-IgG was identified in four patients including those in this report.

**Conclusion:**

Thymoma can be accompanied by anti-CNTN1 AN. Adequate immunotherapies should be considered regardless of the efficacy of thymectomy.

## 1 Introduction

The thymus is the primary lymphoid organ for educating T cells, where positive and negative selection ensures the acquisition of central T cell tolerance (1). Thymomas, neoplasms derived from the epithelial cells of the thymus, are associated with various autoimmune diseases (1, 2). Autoimmune nodopathies (ANs) are immune-mediated neuropathies with disease-specific autoantibodies against membrane proteins located in the nodes of Ranvier and paranodes such as neurofascin 155 (NF155), NF140/186, contactin-1 (CNTN1), and contactin-associated protein 1 (Caspr1) (3). It was recently reported that some patients with anti-CNTN1 AN had paraneoplastic status including thymoma (4). There have been several reports of demyelinating peripheral neuropathy and concomitant thymoma in the literature; however, a causal relationship between the two conditions remains unclear (4–13). This report presents three cases of anti-CNTN1 AN with thymoma and discusses the pathogenesis of thymoma-associated demyelinating neuropathy.

## 2 Methods

### 2.1 Subjects

Cases 1 and 2 were diagnosed with anti-CNTN1 AN at Kyushu University Hospital in 2017 and 2020, respectively. We also included a previously reported case (Case 3) presenting with subacute sensory ataxic neuropathy with thymoma (6), which was seropositive for CNTN1-IgG by further investigation.

### 2.2 Detection of autoantibodies against paranodal proteins

CNTN1-IgG and NF155-IgG in sera from Cases 1 and 2 were evaluated by flow cytometric cell-based assay (CBA) as previously described (14, 15). Caspr1-IgG was screened by enzyme-linked immunosorbent assay (ELISA) using recombinant human Caspr1 protein (R&D Systems, Minneapolis, MN, USA) (16). In Case 3, serum CNTN1-IgG and NF155-IgG were measured at Kindai University by ELISA using commercially available recombinant human CNTN1 and NF155 proteins (Sino Biological, Beijing, China, and R&D Systems, Minneapolis, MN, USA, respectively) as a part of a comprehensive autoantibody evaluation. When positive, IgG subclass analysis was performed as for total IgG detection.

### 2.3 Neurofilament light chain measurement

Serum neurofilament light chain (NfL) was measured in duplicate using a Simoa NF-light™ V2 advantage kit (Quanterix, Billerica, MA, USA) on a Simoa HD-X analyzer following the manufacturer’s instructions and standard procedures.

### 2.4 Literature review

We performed a PubMed search using “neuropathy” and “thymoma” as keywords on 10th October 2024, which identified 65 articles. Then, we reviewed all suitable cases of demyelinating peripheral neuropathy with thymoma by a literature survey.

## 3 Case description

### 3.1 Case 1

A 60-year-old man with a medical history of recurrent invasive thymoma was admitted to our hospital, with the chief complaint of limb weakness. The thymoma was incidentally found on a medical checkup at age 40 years. After thymectomy and postoperative radiation therapy, vitiligo vulgaris appeared on his face and hands. Fourteen and 17 years after the first resection, the thymoma relapsed in the anterior chest wall. Surgical treatments were performed and the histological subtype was type B3 thymoma according to the World Health Organization classification.

The patient presented with paresthesia of both feet spreading to all limbs less than two months before admission. He occasionally had pain similar to sore muscles on the lower back and left lower limb. Bilateral lower limb weakness progressively worsened, resulting in difficulty standing up without the support of the upper limbs. Just before admission, a medical checkup by chest computed tomography (CT) revealed the third recurrence of thymoma in the left intrapleural cavity. Neurological examination showed left-predominant paresthesia, diffuse hyporeflexia, and disturbance of deep sensation in the lower limbs (**Table 1**). Manual muscle testing showed bilateral asymmetric weakness dominantly involving the distal part of left upper and lower limbs. Serum creatine kinase was within normal limits. Although a diagnostic workup found several autoantibodies against double-stranded deoxyribonucleic acid (dsDNA), acetylcholine receptor (AChR), and titin in his serum, no clinical evidence of systemic lupus erythematosus (SLE) or myasthenia gravis (MG) was observed. The Harvey–Masland test did not show any waning in the trapezius and abductor digiti minimi muscles. In addition, he was positive for CV2/collapsin response-mediator protein 5-IgG associated with mixed axonal and demyelinating neuropathy (17). Cerebrospinal fluid (CSF) findings showed albuminocytological dissociation (protein level 210 mg/dL and cell count 5/µL) and an IgG index of 0.66 (normal range: < 0.73). The initial differential diagnoses included paraneoplastic demyelinating neuropathy and multifocal chronic inflammatory demyelinating polyradiculoneuropathy (CIDP). However, a nerve conduction study (NCS) revealed apparent conduction delay in all nerves examined and no rationale for multifocal distribution of demyelination was observed. Brain magnetic resonance imaging (MRI) detected no abnormal findings. Hypertrophy of cervical and lumbosacral nerve roots was not obvious on MR neurography (**Supplementary Figure 1**).

**Table 1:** Clinical features of patients with CNTN1-IgG and concurrent thymoma.

Case Characteristics	Case 1	Case 2	Case 3
Age at onset (years)/Sex	60/Male	75/Male	57/Male
Clinical phenotype^1^	Multifocal	Distal	Sensory-predominant
Onset-to-treatment period (months)	3	12	2
**Neurological findings**			
Cranial nerve involvement	–	Dysgeusia, dysphasia	Dysgeusia
Limb weakness	+	+	–
Muscle atrophy	+	–	–
Tremor	–	–	–
Sensory ataxia	+	+	+
Sensory disturbances	Superficial and deep	Superficial and deep	Superficial and deep
Respiratory failure	–	–	–
Autonomic symptoms	–	–	Constipation, dysuria, and erectile dysfunction
Skin lesions	Vitiligo vulgaris	Pemphigus foliaceus	–
Laboratory findings			
HbA1c (NGSP, %)	5.1	5.7	6.1
M-protein	–	–	–
IgG4 levels (mg/dL)	65	70	NA
Antinuclear antibody	–	1:640	1:160
Double-stranded DNA-IgG	+	+	–
Cardiolipin-IgG	–	+	–
AChR-IgG (nmol/L)	16 (peak)	23.1 (peak)	–
Paraneoplastic antibody panels^2^	CV2/CRMP5, titin	–	–
CSF protein levels (mg/dL)	336 (peak)	309 (peak)	205 (peak)
CSF cell counts (/μL)	7 (peak)	2 (peak)	6
Renal involvement	–	+	–
Nerve conduction studies^3^			
Median nerve	Right/Left	Left	Right/Left
Distal latency (ms)	9.8/8.7	7.3	6.2/6.3
MCV (m/s)	30.6/28.4	42.9	55.0/52.8
CMAP amplitude (mV)	4.1/0.6	4.9	3.9/3.7
F-wave latency (ms)	88.2/135.0	60.2	30.1/31.4
SCV (m/s)	NE/NE	NE	42.6/39.2
SNAP amplitude (μV)	NE/NE	NE	14.7/14.0
Ulnar nerve			
Distal latency (ms)	6.2/5.1	5.6	3.5/4.0
MCV (m/s)	54.7/34.3	47.0	52.9/47.1
CMAP amplitude (mV)	5.6/5.6	4.2	7.8/8.1
F-wave latency (ms)	NE/59.8	58.7	32.3/31.3
SCV (m/s)	NE/NE	NE	44.0/38.7
SNAP amplitude (μV)	NE/NE	NE	25.0/21.0
Tibial nerve			
Distal latency (ms)	7.4/6.5	NE	5.8/5.4
MCV (m/s)	32.6/33.7	NE	50.7/36.8
CMAP amplitude (mV)	6.6/5.0	NE	9.2/12.7
F-wave latency (ms)	NA/102.5	NE	52.5/55.3
Sural nerve			
SCV (m/s)	45.2/42.2	NE	45.2/44.8
SNAP amplitude (μV)	12.4/9.9	NE	5.7/9.5

^1^Defined by the European Academy of Neurology/Peripheral Nerve Society guideline for chronic inflammatory demyelinating polyradiculoneuropathy.

^2^Autoantibodies against Tr (DNER), GAD65, zic4, titin, SOX1, recoverin, Hu, Yo, Ri, PNMA2 (Ma2/Ta), CV2/CRMP5, amphiphysin.

^3^The results before immunotherapy. Normal values: median nerve distal latency < 4.2, MCV > 48, CMAP > 3.5, F-wave latency < 31, SCV > 44, SNAP > 7 (Cases 1 and 2)/> 19 (Case 3); ulnar nerve distal latency < 3.4, MCV > 49, CMAP > 2.8, F-wave latency < 32, SCV > 44 (Cases 1 and 2)/> 47 (Case 3), SNAP > 5 (Cases 1 and 2)/> 18 (Case 3); tibial nerve distal latency < 6.0, MCV > 41, CMAP > 2.9, F-wave latency < 58; sural nerve SCV > 45 (Cases 1 and 2)/> 39 (Case 3), SNAP > 3 (Cases 1 and 2)/> 3.8 (Case 3).

AChR, acetylcholine receptor; CMAP, compound muscle action potentials; CRMP5, collapsin response-mediator protein 5; CSF, cerebrospinal fluid; CNTN1, contactin-1; DNA, deoxyribonucleic acid; DNER, delta notch-like epidermal growth factor-related receptor; GAD, glutamic acid decarboxylase; MCV, motor conduction velocity; NA, not applicable; NE, not evoked; NGSP, national glycohemoglobin standardization program; PNMA2, paraneoplastic antigen Ma2; SCV, sensory conduction velocity; SNAP, sensory nerve action potentials. SOX1, SRY-related HMG-box 1.

**Figure 1:**
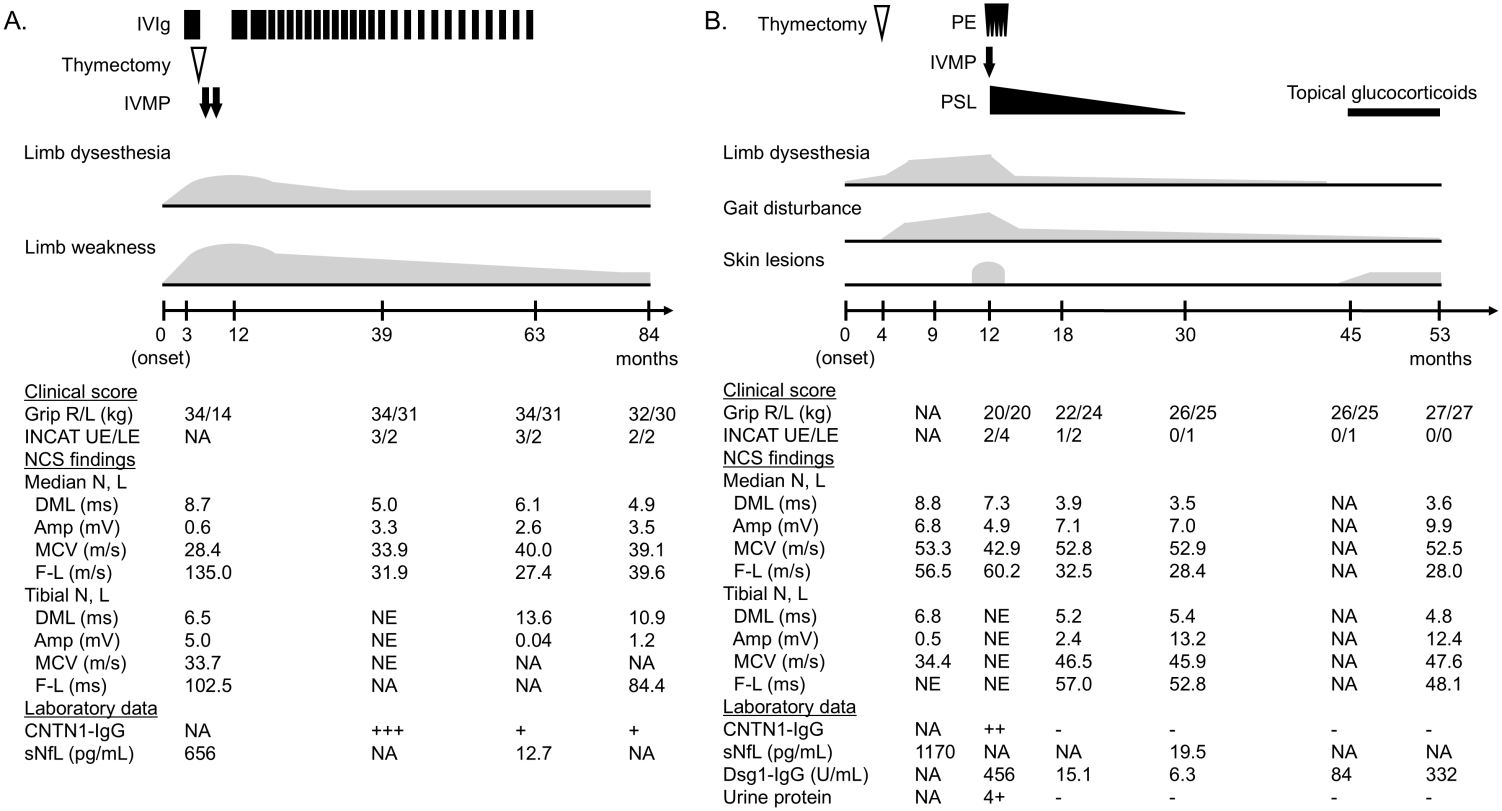
Clinical course of cases 1–3. **(A)** Case 1. **(B)** Case 2. CNTN1-IgG titer was divided into four levels based on the mean fluorescence intensity ratio by flow cytometric cell-based assay: −, +, ++, +++. Normal value of Dsg1-IgG by chemiluminescence enzyme immunoassay is < 20.0 U/mL. Amp, amplitude; CNTN1, contactin-1; DML, distal motor latency; Dsg1, desmoglein 1; F-L, F-wave latency; INCAT, inflammatory neuropathy cause and treatment; IVMP, intravenous methylprednisolone; IVIg, intravenous immunoglobulin; L, left; LE, lower extremities; MCV, motor conduction velocity; N, nerve; NA, not applicable; PE, plasma exchange; PSL, prednisolone; R, right; sNfL, serum neurofilament light chain; UE, upper extremities.

**Figure 2:**
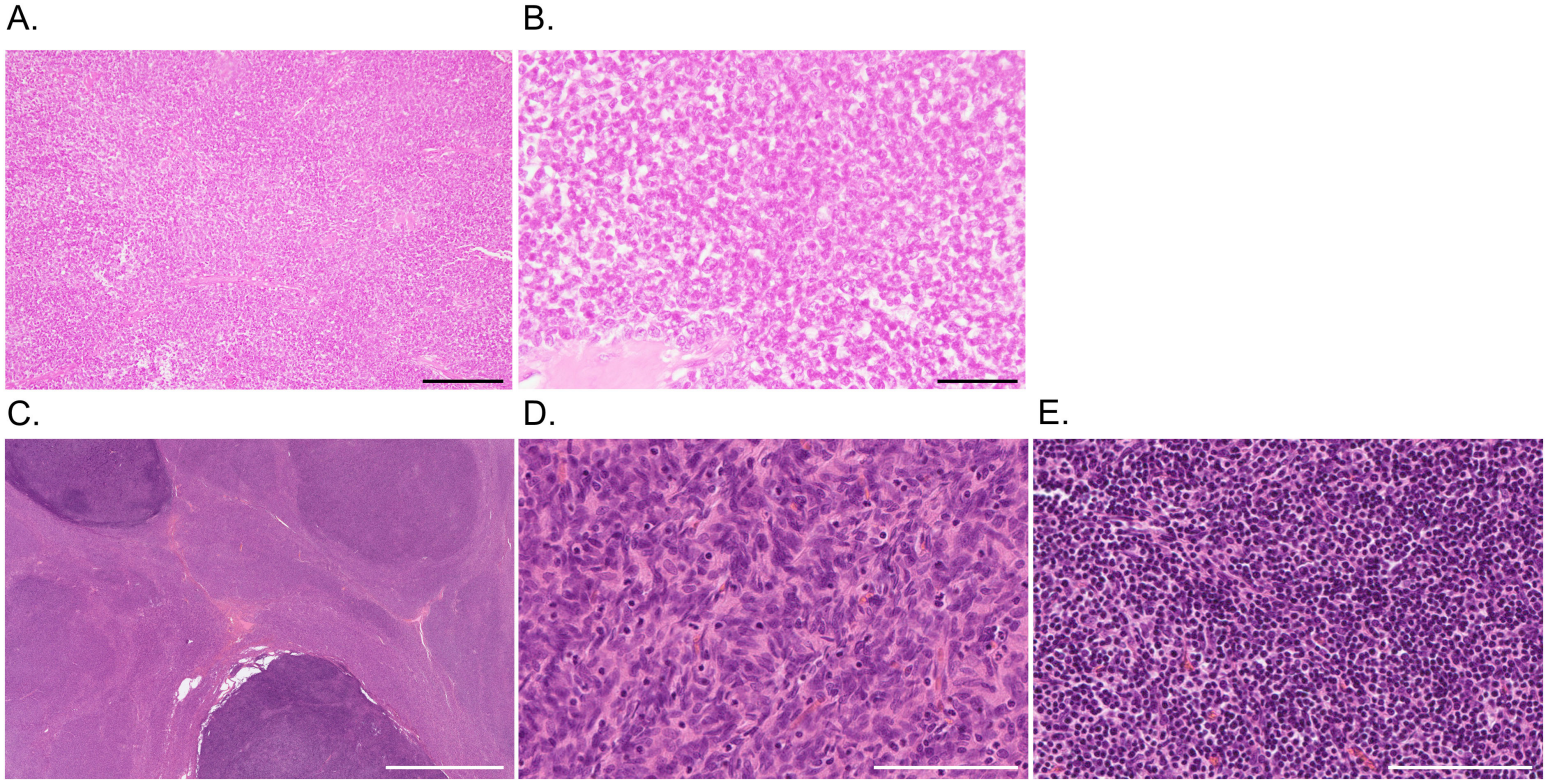
Pathological findings of thymoma. **(A, B)** Case 1. Hematoxylin and eosin staining shows a lobular proliferation of polygonal epithelioid cells in a sheet-like pattern with lymphocytic infiltrate and fibrous septa, indicating type B3 thymoma. **(C-E)** Case 2. At low power magnification **(C)**, the tumor is predominantly composed of eosinophilic areas with nodular darker basophilic portions. In the eosinophilic areas **(D)**, spindle cells proliferate in fascicles with scattered lymphocytes, indicating type A thymoma. The darker component **(E)** is characterized by inconspicuous tumor cells with dense lymphoid tissue, representing type B1 thymoma. Scale bars = 200 μm (A, D, E), 50 μm **(B)**, and 5 mm **(C)**.

Because of the acute deterioration of sensorimotor defects after admission, intravenous immunoglobulin (IVIg, 2 g/kg) was initially administered and slightly improved paresthesia in the left hand (**Figure 1A**). Three weeks later, the patient received the third thymectomy. Microscopic examination revealed a lobular proliferation of polygonal epithelioid cells and infiltration of fibro-adipose tissue in the chest wall, indicating the recurrence of type B3 thymoma (**Figures 2A, B**). Two weeks after thymectomy, the patient received two courses of intravenous methylprednisolone (IVMP, 1000 mg/day for 3 days), which did not affect his symptoms. Treatment was switched to IVIg, followed by maintenance dose IVIg (1 g/kg) once every 1–2 months for 4 years. Neurological symptoms and NCS findings gradually improved (**Figure 1A**). Even after the unexpected discontinuation of IVIg because of acute myocardial infarction, a further improvement in neurological symptoms was achieved with a total Inflammatory Neuropathy Cause and Treatment (INCAT) score of 5 (3 for arms and 2 for legs) at the last administration and 4 (2 for arms and legs) at the last follow-up. Incidentally, vitiligo vulgaris did not improve during treatment.

### 3.2 Case 2

A 75-year-old man initially developed mild dysesthesia in four limbs. Concurrently, thymoma was incidentally found through the evaluation of emphysema. Although AChR-IgG was positive, the patient did not present with clinical or electrophysiological signs of MG. The patient had no other relevant medical or family history. Thymectomy was performed four months later, and the pathological classification was type AB thymoma, characterized by the proliferation of spindle cells admixed with mature lymphocytes, alternating with lymphocyte-rich areas composed of proliferating oval or polygonal epithelial cells (**Figures 2C–E**). After resection of the thymoma, limb dysesthesia and weakness deteriorated acutely resulting in requiring assistance when walking. Intense pruritus and lower limb pitting edema emerged. The patient was transferred to our department for further evaluation.

On admission, the patient presented with mild dysgeusia and dysphagia with no diurnal variation, symmetrical distal limb weakness, superficial and deep sensory disturbances, diffuse hyporeflexia, and ataxic gait with positive Romberg sign (**Table 1**). Tremor was not conspicuous. CSF protein level and IgG index were 309 mg/dL and 0.57, respectively. NCS findings showed evident conduction delay in all nerves tested. Brain MRI was normal and brain N-isopropyl-p-[^123^I] iodoamphetamine single-photon emission CT revealed no definite decreased perfusion. Cervical and lumbosacral MR neurography revealed slightly thickened nerve roots (**Supplementary Figure 1**). On physical examination, skin lesions characterized by edematous erythema, blisters, and erosion affected the head, neck, and four limbs. Mucosal sites were intact. Skin biopsy findings and desmoglein 1 (DSG1)-IgG positivity were consistent with pemphigus foliaceus (**Supplementary Figure 2**). Autoantibodies against DSG3 and bullous pemphigoid antigen 180 were not detected in the serum. The patient was diagnosed with nephrotic syndrome based on hypoalbuminemia (2.4 g/dL) and overt proteinuria (urine protein to creatinine ratio 5.3 g/gCr). His proteinuria selectivity index was 0.16, and urinary occult blood was negative. Hepatitis B virus serological status indicated past infection, which might have been a cause of secondary membranous nephropathy (MN). We did not measure autoantibodies targeting podocyte antigens including phospholipase A2 receptor (PLA2R) and thrombospondin type 1 domain-containing 7A (THSD7A) (18, 19). The patient refused a renal biopsy.

The patient was treated with plasma exchanges followed by IVMP (1000 mg/day for 3 days) and oral glucocorticoids (prednisolone 50 mg/day), resulting in a gradual improvement of neurological findings as well as blisters and leg edema (**Figure 1B**). Three months after treatment initiation, the patient had no skin lesions and could walk without a cane over short distances. Urine protein and serum DSG1-IgG returned to negative six months later. NCS findings also showed remarkable improvement. Eighteen months after the initial treatment, oral glucocorticoids were tapered off, leading to the remission of neurological, skin, and renal symptoms. Fifteen months later, erythema reemerged on the head with an elevation of DSG1-IgG titers. Neurological and renal involvement remained in complete remission with an INCAT score of 0, and there was no evidence of thymoma recurrence. Considering the impact of systemic glucocorticoid administration on a patient with a history of pulmonary tuberculosis, pemphigus was treated with topical glucocorticoids at the last follow-up.

### 3.3 Case 3

The clinical course of Case 3 was documented in detail by Nishijima et al. (6). Briefly, a 57-year-old man presented with a subacute onset of sensory neuropathy characterized by limb paresthesia, gait disturbance with sensory ataxia, autonomic symptoms, and very high CSF protein levels (**Table 1**). Laboratory tests showed no positive autoantibodies such as anti-SS-A and anti-SS-B antibodies. NCS showed sensory conduction abnormalities and prolonged distal motor and F-wave latencies. Approximately three months after neurological onset, invasive thymoma was detected on chest CT. Although IVIg and thymectomy were not effective, daily oral glucocorticoids (prednisolone 30 mg/day) immediately improved his symptoms. The thymoma was pathologically classified as type B3. The patient achieved remission with slight distal paresthesia in the feet. Conduction delay on motor and sensory nerves returned to within normal limits.

### 3.4 Biomarker analysis through the clinical course of disease

Sera from Cases 1 and 2 were positive for CNTN1-IgG by CBA, whereas that of Case 3 was positive by ELISA. The IgG4 subclass of antibodies was predominantly elevated in all cases. Relevant immunoreactivity against CNTN1-IgG was found in the order of IgG1 > IgG2 in Cases 1 and 2. In Case 3, only weak reactivity for IgG1 was detected. Caspr1-IgG was not detected in sera from Cases 1 and 2, and was not tested for in Case 3. None of the three cases were positive for NF155-IgG.

In Case 1, initially suspected as paraneoplastic syndrome or multifocal CIDP, serum autoantibodies against paranodal proteins were not measured until maintenance IVIg was administered, demonstrating CNTN1-IgG positivity. The titer was decreased with the clinical improvement but was constantly positive (**Figure 1A**). The autoantibody status of Case 2 was evaluated before immunotherapy administration. Serum CNTN1-IgG returned to negative three months after treatment initiation, following an improvement in neurological findings (**Figure 1B**). Of note, Case 3 was seropositive for CNTN1-IgG when using a stock serum sample taken before immunotherapy administration. Follow-up measurements using serum from remission were negative for CNTN1-IgG.

sNfL levels of Cases 1 and 2 before immunotherapy were highly elevated (656 pg/mL and 1,170 pg/mL, respectively). At the end of the treatment when clinical remission was achieved, they were markedly decreased to 12.7 pg/mL and 19.5 pg/mL, respectively, which were within the normal ranges based on age-specific reference values (20).

### 3.5 Literature review

Our literature survey identified ten cases of demyelinating peripheral neuropathy with thymoma (4–13). A summary of patients including Cases 1 and 2 is shown in **Table 2**. Ten of eleven patients were male. The mean age at onset of peripheral neuropathy was 56.5 ± 10.6 years (standard deviation). Limb weakness and sensory ataxia were observed in 73% and 71% of patients, respectively. CSF protein levels were elevated in seven of nine patients with CSF data, and our three cases with CNTN1-IgG had levels > 200 mg/dL. Six patients had AChR-IgG including our Cases 1 and 2. Notably, Cases 1 and 2 did not develop symptoms related to MG. Apart from our cases, only two cases have been investigated for CNTN1-IgG positivity: one was positive (4) and the other was negative (9). On the pathological basis of thymoma, WHO classification was available for five patients: type B3 (*n* = 2) and type AB (*n* = 3). Immunotherapies were administered to nine patients and were effective for six. Three poor responders, one with Good’s syndrome and two with malignant thymoma, progressed to death. Another case of malignant thymoma with encephalopathy who did not receive immunotherapy or thymoma treatment resulted in death.

**Table 2:** Cases of demyelinating peripheral neuropathy with thymoma.

Patients	Sex	Age at onset (neuropathy)	Limb weakness	Sensory ataxia	CSF protein levels (mg/dL)	MG symptoms	AChR-IgG	ANA	CNTN1-IgG	Immunotherapy
Case 1	M	60	+	+	210	–	+	–	+	GC, IVIg
Case 2	M	75	+	+	309	–	+	1:640	+	GC, PP
Case 3 (6)	M	57	–	+	205	–	–	1:160	+	GC, IVIg
Deng et al. (7)	M	71	+	–	Normal	NA	NA	–	NA	GC, IVIg
Sawhney et al. (8)	M	40	+	NA	66	–	NA	–	NA	GC, IVIg
Dubey et al. (4)	NA	NA	NA	NA	NA	NA	NA	NA	+	NA
Siles et al. (9)	F	44	+	NA	NA	NA	NA	NA	–	GC
Tajima et al. (5)	M	64	–	+	124	–	–	–	NA	IVIg
Heidenreich et al. (10)	M	51	+	NA	79	+	+	NA	NA	AZA, GC, PP
Perini et al. (11)	M	50	–	NA	NA	+	+	1:80	NA	–
Miller et al. (12)	M	56	+	–	20	+	+	–	NA	GC, PP
Bogousslavsky et al. (13)	M	54	+	+	116	+	+	–	NA	–

Patients	Thymoma	Timing of thymoma detection	Thymoma treatment	Neuropathic status after Tx	Outcome	Remarks
Case 1	Type B3	Prior to neuropathy (neuropathy onset: 20 years after the initial Tx)	Tx, RT	Unchanged (3^rd^ Tx)	Improved	–
Case 2	Type AB	Subsequent to neuropathy	Tx	Deteriorated	Improved	–
Case 3 (6)	Type B3	Subsequent to neuropathy	Tx, RT	Unchanged	Improved	–
Deng et al. (7)	Type AB	Prior to neuropathy (neuropathy onset: 3 years after Tx)	Tx	NA	Dead (PML)	Good’s syndrome
Sawhney et al. (8)	Malignant	Subsequent to neuropathy	–	–	Dead (CA)	–
Dubey et al. (4)	NA	NA	NA	NA	NA	–
Siles et al. (9)	Invasive	NA	NA	NA	Improved	Caspr2-IgG with no evidence of neuromyotonia
Tajima et al. (5)	Type AB	Subsequent to neuropathy	Tx	Slightly improved	Improved	–
Heidenreich et al. (10)	Malignant	Prior to neuropathy (neuropathy onset: 5 years after the initial Tx)	Tx, RT, Cx	NA	Dead (CPA)	Neuromyotonia and encephalopathy with VGKC-IgG
Perini et al. (11)	Lymphoepithelial	Subsequent to neuropathy	Tx	Deteriorated	Improved	Neuromyotonia
Miller et al. (12)	Invasive	Subsequent to neuropathy	Tx, RT	Improved	Improved	–
Bogousslavsky et al. (13)	Malignant	Subsequent to neuropahty	–	–	Dead (CPA)	Encephalopathy

AChR, acetylcholine receptor; ANA, antinuclear antibody; AZA, azathioprine; CA, cardiac arrest; Caspr2, contactin-associated protein 2; CNTN1, contactin-1; CPA, cardiopulmonary arrest; GC, glucocorticoid; CSF, cerebrospinal fluid; Cx, chemotherapy; F, female; IVIg, intravenous immunoglobulin; M, male; MG, myasthenia gravis; NA, not applicable; PML, progressive multifocal leukoencephalopathy; PP, plasmapheresis; RT, radiation therapy; Tx, thymectomy; VGKC, voltage-gated potassium channel.

## 4 Discussion

In this study, we presented three cases of CNTN1-IgG4 and concurrent thymoma in detail. CNTN1-IgG4 is characterized by elderly onset, acute or subacute disease progression, distal-dominant limb weakness, sensory ataxia, very high CSF protein levels, apparent conduction delay on NCSs, and a poor response to IVIg (4, 21), consistent with the neurological findings of all cases in this study. In Cases 1 and 3, immunotherapies were started within a few months after neurological onset, indicating subacute disease progression. Of interest, Case 2 demonstrated acute deterioration after thymectomy. Despite the disease severity at nadir, their clinical and electrophysiological states achieved a drastic improvement by the combination of first-line therapies for CIDP. Similarly to Case 1, some patients have been reported to show partial or good response to IVIg (4, 21, 22). The inhibition of complement activation triggered by coexisting non-IgG4 autoantibodies might lead to the efficacy (23). In addition, IVMP might have contributed, at least to some extent, to controlling the disease activity; the therapeutic effect was not evident at the severity peak of the disease course. In particular, daily glucocorticoids were effective in Cases 2 and 3 (21). Although rituximab has been reported to be effective in anti-CNTN1 AN (22), it is not currently covered by medical insurance systems in Japan. CNTN1-IgG titers and sNfL levels paralleled the clinical improvement in individuals, suggesting its clinical utility for monitoring disease activity and treatment responses.

Case 2 was complicated with nephrotic syndrome, which improved in parallel with neuropathy and CNTN1-IgG status. MN often occurs in association with anti-CNTN1 AN (24, 25). Primary MN is caused by autoantibodies against podocyte proteins, resulting in the formation of immune deposits on the outer aspect of the glomerular capillary wall. Many target antigens including PLA2R and THSD7A have been identified (18, 19, 24), although their comprehensive screening was not performed in this study. However, CNTN1 protein is also expressed in healthy kidney glomeruli, and immune complexes containing CNTN1 are found in affected glomeruli (25). Lupus nephritis might be a differential diagnosis based on several autoantibodies including antinuclear antibody, dsDNA-IgG, and cardiolipin-IgG, which fulfill the diagnostic criteria for SLE (26, 27). However, the clinical manifestation was not typical for SLE, considering age, sex, and normal serum complement levels.

As far as we know, there has been only one case of anti-CNTN1 AN with thymoma, which was detected within six months of neuropathy onset (4). However, detailed clinical information was not documented in the article. Some cases with anti-CNTN1 AN had concomitant malignancies including breast cancer, plasmacytoma, lymphoma, and colon adenocarcinoma (4, 9). Despite many reports of anti-NF155 AN, there have been no cases associated with thymoma. Thymoma might be a coincidental lesion and the elderly onset in anti-CNTN1 AN compared with anti-NF155 AN might explain the higher comorbidity of neoplasms (28). However, in Case 2, neurological findings worsened after thymectomy, suggesting an important role for thymic function in regulating immune responses in the pathology of anti-CNTN1 AN. Our literature survey indicated no precise relationship between demyelinating peripheral neuropathy and thymoma, possibly because of the small number of cases. Clinical characteristics were heterogeneous except for male predominance and middle-aged onset. Some cases were complicated with other neurological disorders including MG, neuromyotonia, and encephalopathy. Although the efficacy of individual immunotherapy was undetermined because of the different protocols used and time points administered, most cases of demyelinating peripheral neuropathy with non-malignant thymoma responded well to immunotherapies. All patients with malignant thymoma progressed to death. However, although there was a low number of antibody-tested cases, it was found that CNTN1-IgG was positive in four patients, suggesting autoantibodies might be associated with thymoma-related demyelinating neuropathy.

Several mechanisms have been proposed to explain the pathological link between thymoma and autoimmunity based on the positive and negative selection of T-lymphocytes in the thymus. The “escape theory” hypothesizes that immature thymoma-derived T-lymphocytes escape from the disorganized tumor environment without receiving self-tolerance in the thymic medulla (29). The “genetic theory” postulates that an abnormal cortical environment in thymomas undergoes rapid proliferation and genetic changes, which generates self-reactive lymphocytes (30, 31). The “autoimmune regulator (AIRE) theory” describes a deficiency in thymomas, a failure in negative selection, leading to the release of autoreactive T-lymphocytes into peripheral tissues (32). In addition, as in Case 2, some patients develop autoimmune diseases after thymectomy, which challenges these current theories and implies a more complex defect in T-lymphocyte maturation. Sera from all cases in the present study included various autoantibodies, probably reflecting indiscriminate immune responses associated with thymoma. Of interest, two of three patients had AChR-IgG without any symptoms of MG. The prevalence of subclinical MG with AChR-IgG in thymomas ranged between 11% and 25% (33–35). A retrospective single-center observational study reported that 91% of patients with subclinical MG became symptomatic within 6 years after thymectomy (35). Case 1 is less likely to develop MG based on the follow-up period of more than 20 years after thymectomy. Although Case 2 has not developed MG symptoms 4 years after thymectomy, we cannot exclude the possibility of it being masked by immunotherapy.

Recently, the concept of autoimmune diseases mediated by antigen-specific autoantibodies of the IgG4 subclass was proposed as IgG4 autoimmune diseases (IgG4-AIDs) (36). Target transmembrane or extracellular antigens have been identified in multiple organs including the central and peripheral nervous systems, skin, kidney, and hematological system (37). IgG4 autoantibodies in IgG4-AIDs are directly pathogenic by blocking protein–protein interactions (37). CNTN1-IgG and DSG1-IgG, both of which are predominantly of the IgG4 subclass, were proven to be pathogenic by passive-transfer animal models (38, 39). Of note, Case 2 concurrently developed several IgG4-AIDs in different organs, suggesting that individual IgG4-AID may share a common underlying pathomechanism. IgG4 subclass predominance in IgG4-AIDs may not be driven by the overall tendency to develop IgG4 responses but rather by antigen-specific selectivity based on normal levels of serum IgG4 concentrations and numbers of circulating IgG4-positive plasma cells and B cells (40). There is a strong association with specific HLA class II alleles in IgG4-AIDs, which can predispose individuals to disease by the recognition of autoantigens by T cells, which directs B cell development and cytokine production (41). The HLA restriction for anti-CNTN1 AN is unknown and should be elucidated in the future. Notably, IgG4-AIDs responded well to rituximab, often resulting in long-term remission and negative conversion of autoantibodies (42–44).

Our study has several limitations. First, immunohistochemical analysis for thymoma were not performed. Further exploration, including immune cell subsets and AIRE expressions would provide insights for the mechanisms of immune tolerance failure in the thymus. Second, we could not evaluate the pathology of nephrotic syndrome because of the refusal for biopsy. Tissue-based data confirming kidney-neural involvement mediated by CNTN1-IgG would strengthen systemic immunological disruption after thymectomy. Finally, CNTN1-IgG was measured in a part of previous reports describing demyelinating peripheral neuropathy with thymoma. Clinical data were limited and most cases showed low CSF protein levels, suggesting a reduced likelihood of CNTN1-IgG positivity. Further research, including additional cases, is needed to confirm the potential link between CNTN1 autoimmunity and thymoma involvement.

In conclusion, we presented detailed clinical information, pathological findings, and biomarker analysis of three patients with anti-CNTN1 AN and concurrent thymoma. Anti-CNTN1 AN can be regarded as a thymoma-related autoimmune disease. A case complicated with pemphigus foliaceus may provide some clues to elucidate a common underlying etiology of IgG4-AIDs.

## Data Availability

The datasets presented in this article are not readily available. Requests to access the datasets should be directed to Hidenori Ogata, ogata.hidenori.565@m.kyushu-u.ac.jp.
