# Supplementary material for: Anti-contactin-1 autoimmune nodopathy with thymoma: case report and literature review
Source: Front Immunol. 2025 Sep 9;16:1662299. doi: 10.3389/fimmu.2025.1662299 (PMC12455620; doi:10.3389/fimmu.2025.1662299)
Supplement: Supplementary file 1 [file Table1.docx]

Supplementary Material

# Supplementary Figures

**
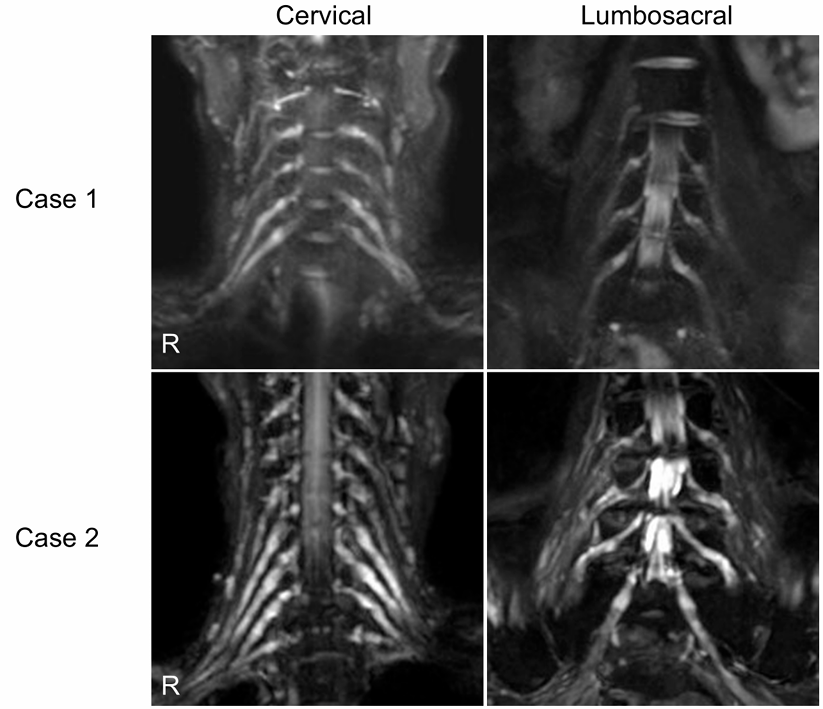
**

**Supplementary Figure 1.** **Magnetic resonance neurography.** Case 2 shows slightly symmetric hypertrophy of the cervical and lumbosacral spinal roots.


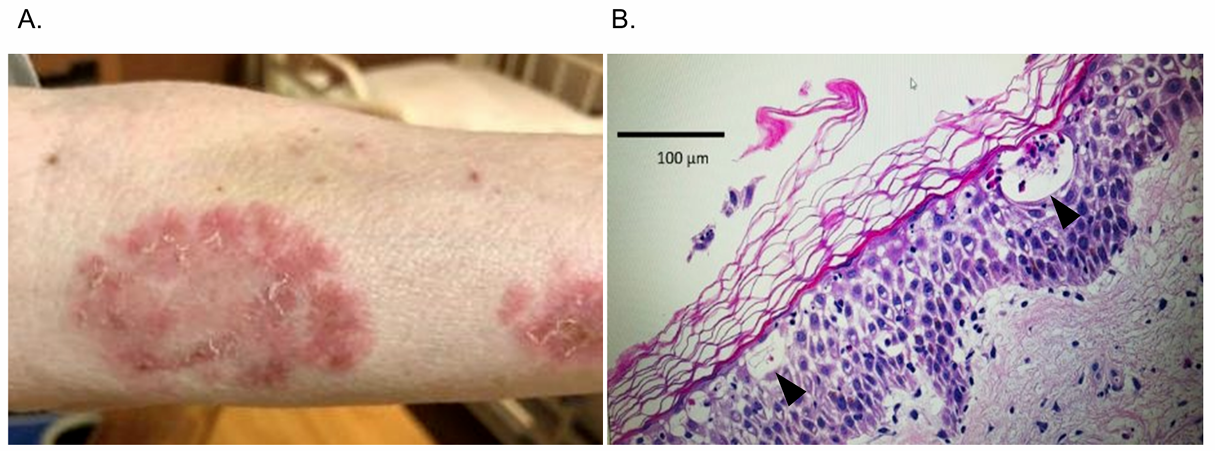


**Supplementary Figure 2.** **Representative images of skin lesions and biopsy findings.** (A) Case 2 developed annular-shaped edematous erythema and blisters on the forearms. (B) Hematoxylin and eosin staining indicated spongiosis, neutrophilic or eosinophilic infiltration, and vacuolar degeneration (arrowheads) accompanied by small subcorneal bulla at the epidermis.
